# Supplementary material for: Historical Text Image Enhancement Using Image Scaling and Generative Adversarial Networks
Source: Sensors (Basel). 2023 Apr 14;23(8):4003. doi: 10.3390/s23084003 (PMC10142040; doi:10.3390/s23084003)
Supplement: Supplementary file 1 [file sensors-23-04003-s001.zip › sensors-2211075-supplementary.pdf]

Supplementary Materials:

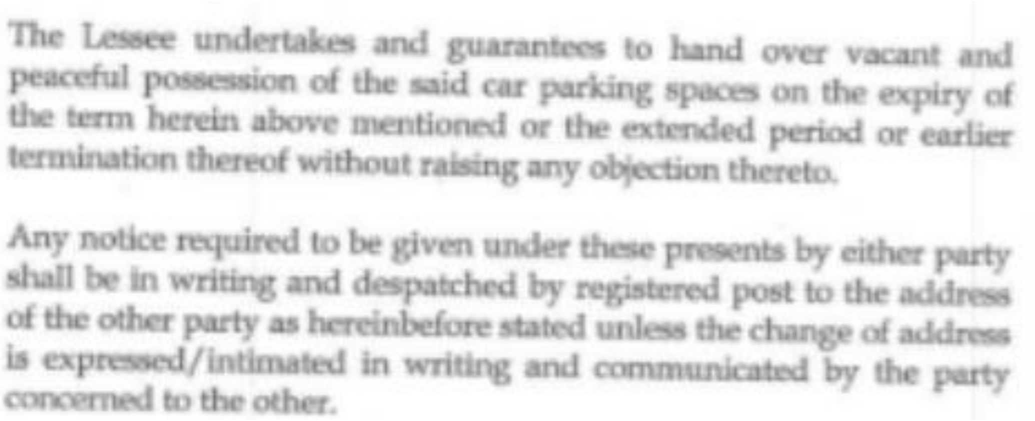

The Lessee undertakes and guarantees to hand over vacant and peaceful possession of the said car parking spaces on the expiry of the term herein above mentioned or the extended period or earlier termination thereof without raising any objection thereto.

Any notice required to be given under these presents by either party shall be in writing and despatched by registered post to the address of the other party as hereinbefore stated unless the change of address is expressed/intimated in writing and communicated by the party concerned to the other.

Figure S1. Image fading (image source [5]).

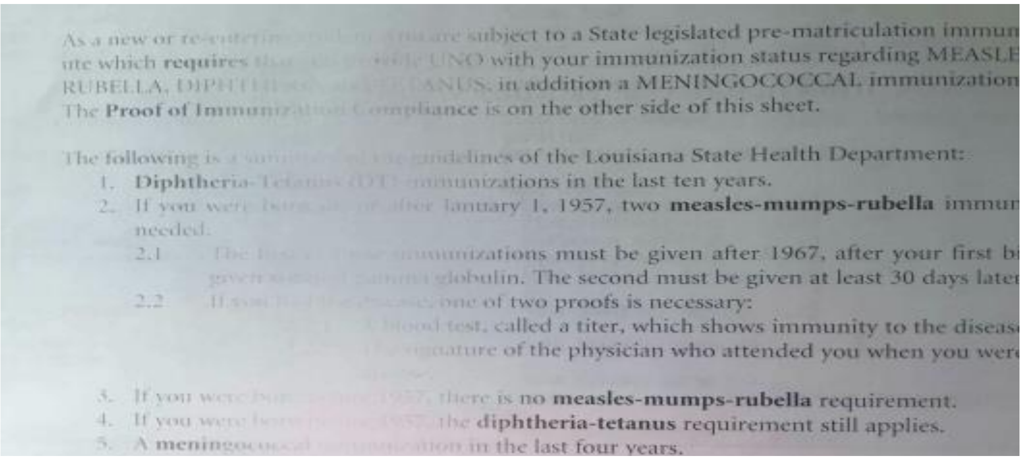

As a new or [illegible] are subject to a State legislated pre-matriculation immun[illegible]
ute which **requires** [illegible] UNO with your immunization status regarding MEASLE[illegible]
RUBELLA, DIPHT[illegible] TANUS, in addition a MENINGOCOCCAL immunization
The **Proof of Immun**[illegible] Compliance is on the other side of this sheet.

The following is [illegible] guidelines of the Louisiana State Health Department:

1. **Diphtheria**-[illegible] immunizations in the last ten years.
2. If you were [illegible] after January 1, 1957, two **measles-mumps-rubella** immun[illegible] needed.
   - 2.1 The [illegible] immunizations must be given after 1967, after your first bi[illegible] [illegible] globulin. The second must be given at least 30 days later
   - 2.2 If [illegible] one of two proofs is necessary:
     - [illegible] blood test, called a titer, which shows immunity to the diseas[illegible]
     - [illegible] signature of the physician who attended you when you wer[illegible]
3. If you were [illegible] there is no **measles-mumps-rubella** requirement.
4. If you were [illegible] the **diphtheria-tetanus** requirement still applies.
5. A **meningo**[illegible] in the last four years.

Figure S2. Severe fading making text illegible.

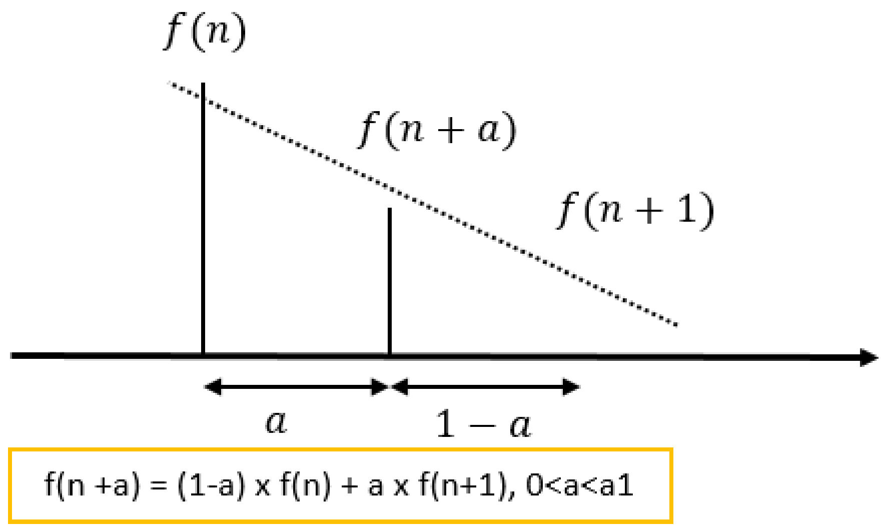

Figure S3. Interpolation example (when a=0.5, we simply have the average of two parameters).
